# Good intentions and the costs of inaction: Financial protection in Austria^☆^

**DOI:** 10.1016/j.hpopen.2025.100159

**Published:** 2025-12-16

**Authors:** Christoph Stegner, Thomas Czypionka

**Affiliations:** Institute for Advanced Studies, Vienna, Austria

**Keywords:** Financial protection, Catastrophic health expenditure, Out-of-pocket payments, Austria, Health policy

## Abstract

Understanding the financial strain of health care costs borne by households is crucial for assessing the equity and affordability of a health system. Building on an already generous system, Austrian health policy has strived over decades to include more and more people in its Social Health Insurance Schemes and to find ways to lower individual financial burden. Using data from the Austrian household budget survey for the years 2004/05, 2009/10, 2014/15 and 2019/20, this study investigates whether these efforts have been successful. Analyzing expenditures on various health care types, including medicines, medical products, outpatient care, dental care, diagnostic tests and inpatient care, our study aims to determine how the percentage of households experiencing catastrophic health expenditure (CHE) according to WHO definitions has changed over time. Logistic regression analyses were carried out to identify factors associated with CHE. The results reveal an increase in the prevalence of CHE from 2.1 % in 2004/05 to 3.6 % in 2019/20. Across all survey rounds, at least 60% of households experiencing CHE belonged to the poorest consumption quintile. Age, sex, educational attainment and employment status of the head of the household emerged as factors associated with CHE from the regression analysis. The observed rise in CHE is surprising given Austria’s generous health system and the introduction of policies during the study period aimed at expanding the breadth and depth of coverage. It serves as an example for other countries that failing to tackle underlying structural problems in the healthcare system may counteract financial protection policies.

## Highlights

• Austrian health policy has extended breadth and depth of coverage over decades.
• Notwithstanding, the share of Austrian households facing catastrophic health expenditure has increased.
• The main drivers of this unfavorable trend are outpatient services and diagnostics.
• This may be owed to inaction to reform the system as a whole.

## 1 Introduction

Ensuring equitable access and financial protection of individuals in case of health problems are primary goals of most healthcare systems [1]. A major obstacle to these goals are out-of-pocket payments, resulting from gaps in any of the three dimensions of healthcare coverage [2], i.e. who is covered (breadth), which services are covered (depth) and to what extent the costs are borne by a third party payer (height). In general, more affluent countries can afford better coverage, and underperforming in this area is amenable to policy changes [3].

Out-of-pocket expenditure (OOPE) of households and their social impact have been analyzed together with unmet needs across the WHO European Region in recent studies, based on quantitative information from household consumption surveys and comprehensive qualitative information [4], [5]. These studies could identify several dynamics and areas that contribute to the problem. In general, poorer households and households with older members are more likely to experience financial hardship due to health-related expenditures. One reason is the existence of gaps in population coverage (breadth), but also gaps in covered services (depth) and user charges (height). The breadth of population coverage is significantly inversely correlated with financial hardship and unmet medical needs, but even high-income countries exhibit gaps with respect to generally small but vulnerable groups like unregistered migrants. OOPE occurring in terms of lack of services covered and user charges is mainly found in outpatient medicines, dental care and primary care services. Policies to extend coverage and to limit the impact of user charges have been found to have significant impact on reducing the number of households experiencing financial hardship.

Austria was also part of these studies and fared generally well in the indicators used, with some room for improvement [6]. In this paper, we analyze the data for this country more deeply. Austria is a federal republic with roughly 9 million inhabitants. It is affluent even in comparison to other EU member states, with a GDP per capita of 44,065 EUR PPP and an unemployment rate of 4.8 % in 2022 [7]. Its health system follows the Bismarckian tradition and – as one of very few – has never introduced competition between health insurance carriers. Another rather rare attribute is the strong role its nine federal states play in hospital care, resulting in considerable fragmentation of care [8]. Physician care outside hospitals comprises both GPs and specialists, still mainly in single practice [9]. Due to the unusually high number of stakeholders with decision powers, the system remains fragmented and the speed of reforms is very slow [10].

Health policy has been dedicated to reduce financial hardship for decades. In terms of population coverage, continuous efforts have been made to bring more and more people under health insurance coverage, with the relevant government body stepping in to pay contributions. E.g. for asylum seekers, the federal government pays contributions, while for the unemployed, it is the unemployment insurance agency.

In terms of depth of coverage, Austria already has a quite extensive coverage policy and gives everyone the means to appeal if services are not covered. Several mechanisms have been introduced over time to curb the amount of user charges, with a general exemption for the poor and a cap of 2 % of net income for prescription drug copayments introduced in 2008 (“Rezeptgebührenobergrenze”, REGO). Health policy has reaffirmed its pledge to “strengthen the benefits in kind principle” in the course of the two latest financial equalization agreements (2017–2023 and 2024–2028) between the federal and state governments. This phrase expresses the intention to increase the number of providers with a direct contract with SHI (and whose services are therefore benefits in kind), as opposed to people seeking care with purely private providers, receiving only a subsidy.

Nonetheless, several studies have shown that equitable access to healthcare services and financial protection are far from perfect. An earlier study of the Austrian household budget Survey of 2009/10 showed an inequitable income redistribution due to OOPE [11]. A study on the same dataset regarding dental care revealed that OOPE for this type of care increased with household size, age, income and education, but not with private insurance [11]. This could reflect the fact that dental services are covered rather well in comparison to other countries. Another study finds an unequal regional distribution of GPs and specialists holding a contract with SHI, which worsened between 2002 and 2014 [12]. This could contribute to financial hardship of households, as they may be forced to seek out care in the private sector.

Regarding prescription medicines, a study using the Austrian Health Interview Survey for 2006/07 finds that individuals with higher incomes tend to consume more non-prescribed medicines, while individuals with lower incomes and lower education levels were more likely to take prescription medicines, but also encounter higher rates of polypharmacy [13]. Finally, a larger study on the Austrian household budget surveys of 2004/05, 2009/10 and 2014/15 revealed an increase in the share of households with catastrophic health expenditure (albeit only to 3.2 % in 2014/15) and identified dental care expenditures, medical products and outpatient medicines to be the major causes of financial hardship [6]. The report also recommended to extend the cap on user charges for prescription medicines to all types of care as an effective means to turn the trend around.

Given that, on the one hand, this previous research may or may not indicate a turn for the worse, and on the other hand continuous policy emphasis lies on improving financial protection, this study attempts to answer three questions: Were the numbers in 2014/15 a one-time exception or is there a worsening trend? Has the composition of OOPE changed over time, especially in households with catastrophic health expenditure (CHE)? What were the main determinants of CHE?

To answer these questions, we will first briefly explain the methodology and data and then present descriptive and regression results from four waves of the Austrian household budget survey. Subsequently, we will discuss our findings in the light of reforms implemented in the past two decades and put the findings in an international context. We will finally draw conclusions and attempt to give policy recommendations that go beyond the case.

## 2 Data and methods

This study utilizes data from the Austrian household budget survey, conducted by Statistics Austria every five years. In particular, the four most recent survey rounds of 2004/05 [14], 2009/10 [15], 2014/15 [16] and 2019/20 [17] were taken for analysis. These surveys are nationally representative and collect comprehensive socio-economic characteristics of households along with detailed information on household spending. The Classification of Individual Consumption by Purpose (COICOP) is used to capture household expenses.

To assess financial protection against health expenditures, this study applies the methodology developed by the WHO Regional Office for Europe [4] for identifying households experiencing CHE. Households are identified as experiencing CHE if they spend 40 % or more of their capacity to pay on OOPE. Capacity to pay is defined as total household expenditure minus subsistence spending on food, housing and utilities which is the average expense on these items between the 25th and 35th percentiles of the household consumption distribution. The OECD equivalence scale is used to adjust for household composition.

In addition to the 40 % threshold, main results are also reported for thresholds of 20 %, 25 % and 30 %. Furthermore, for the 20 % and 40 % thresholds the distribution of CHE among consumption quintiles as well as logistic regression analyses are provided to identify associated factors of CHE. The regression models include socioeconomic and demographic variables such as age, sex, employment status and educational attainment of the head of the household and household size. Adjusted odds ratios (aORs) are calculated to quantify the association between these factors and the odds of experiencing CHE.

Finally, OOPE of households experiencing CHE (40 % threshold) is categorized into the following types of health care: 1) medicines, 2) medical products, 3) outpatient care, 4) dental care, 5) diagnostic tests and 6) inpatient care. This categorization provides insights into the specific drivers of CHE and was chosen based on prior research [5].

## 3 Results

### 3.1 Sociodemographic characteristics

Table 1 shows the sociodemographic characteristics of all households and those exceeding the 40 % and 20 % thresholds for CHE across the 2019/20 survey period. Male-headed households, made up 58 % of the total sample in 2019/20, down from 64 % in 2004/05. In contrast, female-headed households accounted for 42% of the total sample, but were disproportionately represented among those exceeding the 20 % threshold.

**Table 1:** Sociodemographic characteristics of all households and those exceeding the 40% and 20% threshold of CHE.

	2019/20			
	Total (n)	Total (%)	40 % threshold (%)	20 % threshold (%)
Male	3102	58	58	52
Female	2217	42	42	48
0–59 years	3459	65	51	47
60–69 years	848	16	11	13
70 + years	1012	19	38	40
Single	1441	27	16	21
Married	2575	48	55	49
Other	1302	24	29	30
Employed	3157	59	40	39
Unemployed	238	4	7	5
Retired	1716	32	48	51
Other	208	4	5	5
Lower education	745	14	32	23
Secondary education	3518	66	60	65
Higher education	1056	20	8	12
High population density	1820	34	42	35
Intermediate population density	1635	31	27	30
Low population density	1865	35	31	35
Single-person household	1999	38	31	37
2-person household	1636	31	29	34
3/4-person household	1358	26	27	23
5 + person household	326	6	13	7
0 children up to 13 years	4351	82	82	87
1 or 2 children up to 13 years	852	16	13	11
3 or more children up to 13 years	116	2	6	2

The share of households headed by individuals aged 70 and older was 19 % in 2019/20. However, their representation among households exceeding both CHE thresholds was about double their share in the overall sample. In older surveys (2004/05), this overrepresentation was up to 3 times higher, although it remained consistently about twice as high in the last two surveys. Similarly, households headed by retirees were represented more frequently in the CHE thresholds than in the overall sample. Their share in the overall sample was about a third in 2019/20, but approximately 50 % of households with CHE at either threshold were headed by retirees.

The share of households headed by individuals of lower education levels was 14 % in 2019/20. Over time, that share decreased. Also, they had shares exceeding the CHE thresholds compared to the overall sample.

There was a roughly even distribution of households living in high, intermediate or low population density areas in 2019/20. Compared to 2004/05, fewer people lived in high population areas. Most households (82 %) had no children up to 13 years. The share of single-person households rose slightly, from 34 % in 2004/05 to 38 % in 2019/20. The remaining households were almost evenly split between two-person households and those with three or more members. Generally, in characteristics pertaining to population density, household size and number of children, overall shares were closer to the threshold shares compared to characteristics pertaining to sex, age, occupational status and education.

### 3.2 Catastrophic health expenditure (CHE)

**Fig. 1:**
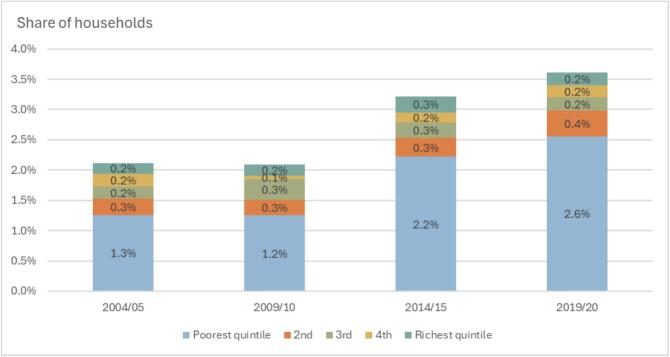
Share of households with CHE at the 40 % threshold.

Fig. 1 presents the shares of households suffering from CHE at the 40 % threshold along with their distribution across consumption quintiles over all four survey waves. A similar figure on the 20 % threshold is available in Supplementary Fig. S2 while more detailed data, including on the 25 % and 30 % thresholds and distribution of households with CHE across consumption quintiles, can be found in Supplementary Tables S3 and S4.

In 2004/05, the incidence of catastrophic health expenditure stood at 2.1 % of households, increasing to 3.6 % in 2019/20, at the 40 % threshold. A significant portion of this increase occurred between the 2009/10 and 2014/15 surveys, where CHE rose from 2.1 % to 3.2 % of households. The distribution of CHE among consumption quintiles reveals that the poorest consumption quintile consistently faced the highest burden. In 2004/05, 59.5 % of households experiencing CHE (or 1.3 % of all households) were from the poorest consumption quintile, while only 8.3 % (or 0.2 % of all households) were from the richest consumption quintile. By 2019/20, the share of CHE affecting the poorest quintile had increased to 70.8 % (or 2.6 % of all households), with the richest quintile’s share declining to 5.8 % (or 1.2 % of all households).

At the 20 % threshold, the evolution of CHE followed a similar trend. In 2004/05, 7.0 % of households experienced CHE, rising to 10.8 % in 2019/20. Again, most of the increase took place between the 2009/10 and 2014/15 survey iterations, with CHE increasing from 7.5 % to 9.3 %. The composition of CHE by quintiles also shows a concentration of CHE among the poorest households, although less so than at the 40 % threshold. In 2004/05, 40.7 % of households facing CHE (or 2.9 % of all households) were from the poorest quintile, which increased to 44.1 % (or 4.8 % of all households) in 2019/20. Conversely, the richest quintile saw a decrease in their share of CHE from 15.7 % (or 1.1 % of households) to 11.7 % (or 1.3 % of all households) over the same period.

Supplementary Tables S4 and S5 also show the results for the COVID-19 period in 2020 (March 2020 – June 2020) and the complete data set (June 2019 – June 2020). In the COVID-19 period, 6.3 % and 15.7 % of households were affected by CHE at the 40 % and 20 % threshold, respectively. Considering the whole period, the share of affected households decreased to 4.2 % and 12.0 %, respectively. While those numbers are elevated compared to the pre-COVID-19 period, the distribution of affected households among consumption quintiles is comparable to the pre-COVID-19 period.

### 3.3 Associated factors

Table 2 shows the adjusted odds ratios (aORs) for selected variables obtained from logistic regression analyses for the 20 % and 40 % CHE thresholds across four survey waves. The full regression results can be found in Supplementary Table S5. The variance inflation factor was consistently below 5, but age and retirement are naturally correlated. Age consistently emerged as a key factor, with households headed by individuals aged 70 and older facing notably higher odds of CHE across all years and thresholds. For instance, in 2004/05, this group had 4.61 times higher odds (p < 0.01) of CHE at the 20 % threshold and 4.31 times higher odds (p < 0.01) at the 40 % threshold compared to those under 60 years old. This pattern persists over time, although the magnitude slightly decreases in subsequent survey rounds (e.g., aOR = 3.21, p < 0.01, at the 20 % threshold in 2019/20).

**Table 2:** Adjusted Odd Ratios of factors associated with CHE from logistic regression analysis.

	2004/05		2009/10		2014/15		2019/20	
Variables	20 % threshold	40 % threshold	20 % threshold	40 % threshold	20 % threshold	40 % threshold	20 % threshold	40 % threshold
Female (ref = male)	1.282*	1.560**	1.419***	1.023	1.469***	1.526*	1.324***	0.994
	(0.163)	(0.347)	(0.188)	(0.262)	(0.175)	(0.343)	(0.143)	(0.186)
60–69 years old (ref = 0–59 years old)	2.612**	0.976	1.613	2.463	1.542**	1.336	1.118	0.853
	(1.194)	(0.386)	(0.474)	(2.103)	(0.323)	(0.525)	(0.292)	(0.419)
70 + years old	4.610***	4.313***	3.001***	6.085**	2.900***	2.350**	3.205***	2.379*
	(1.823)	(1.638)	(0.881)	(5.074)	(0.624)	(0.825)	(0.898)	(1.126)
Unemployed (ref = employed)	0.459**	0.672	1.300	1.084	1.265	2.854***	1.717**	2.016*
	(0.158)	(0.345)	(0.390)	(0.545)	(0.335)	(1.112)	(0.429)	(0.731)
Retired	0.897	1.537	1.203	1.056	1.302	2.705***	1.436	1.696
	(0.418)	(0.573)	(0.341)	(0.868)	(0.272)	(0.954)	(0.374)	(0.758)
Other occupation	1.124	2.029*	1.615	1.260	1.728**	3.197**	2.206***	2.160*
	(0.355)	(0.836)	(0.593)	(0.786)	(0.446)	(1.459)	(0.562)	(0.997)
Secondary education (ref = lower education)	0.874	0.970	0.919	0.690*	0.790*	0.639**	0.734**	0.467***
	(0.137)	(0.246)	(0.130)	(0.151)	(0.104)	(0.140)	(0.107)	(0.110)
Higher education	0.712	0.738	0.732	0.270***	0.653**	0.439**	0.483***	0.202***
	(0.165)	(0.322)	(0.141)	(0.113)	(0.125)	(0.162)	(0.0955)	(0.0749)
Prefer not to say	0.992	1.288						
	(0.231)	(0.543)						
Constant	0.0501***	0.00810***	0.0531***	0.0174***	0.0564***	0.0114***	0.0780***	0.0341***
	(0.0123)	(0.00316)	(0.0123)	(0.00697)	(0.0124)	(0.00469)	(0.0168)	(0.0130)
Observations	8,394	8,394	6,531	6,531	7,158	7,158	5,319	5,319

std. err. in parentheses.

*** p < 0.01, ** p < 0.05, * p < 0.1.

Sex was also identified as an associated factor, though it reached statistical significance less often than age. For example, in 2014/15 households headed by females were associated with higher odds of experiencing CHE at the 20 % (aOR = 1.47, p < 0.01) and 40 % (aOR = 1.53, p = 0.06) threshold.

In recent survey waves, employment status and educational attainment of the head of the household have become increasingly relevant. Unemployed individuals showed significantly higher odds of CHE, particularly in 2014/15 (aOR = 2.85, p < 0.01, at the 40 % threshold) and 2019/20 (aOR = 2.02, p = 0.053, at the 40 % threshold). Conversely, higher educational attainment was associated with lower odds of experiencing CHE compared to lower educational attainment (e.g., at the 40 % threshold in 2014/15 (aOR = 0.44, p = 0.026) and 2019/20 (aOR = 0.20, p < 0.01)).

### 3.4 OOPE on types of health care

Table 3 provides a breakdown of OOPE among households exceeding the 40 % CHE threshold across different types of health care over time. In 2004/05, the highest share of OOPE for households with CHE was on dental care (27.7 %), followed by medical products (23.8 %), medicines (17 %), outpatient care (16.1 %), diagnostic tests (9.7 %) and inpatient care (5.9 %).

**Table 3:** Breakdown of OOPE by type of health care among households affected by CHE (40% threshold).

	Medicines	Medical products	Outpatient care	Dental care	Diagnostic tests	Inpatient care
2004/05	17.0	23.8	16.1	27.7	9.7	5.9
2009/10	11.2	37.2	8.9	25.7	9.7	7.3
2014/15	13.9	12.3	15.4	26.9	16.3	15.1
2019/20	11.0	14.7	23.2	25.1	17.6	8.4

By 2009/10, spending on medical products increased to 37.2 %, while outpatient care and medicines showed decreases in their shares. In 2014/15, inpatient care, diagnostic tests and outpatient care all saw a notable rise in OOPE. Meanwhile, medical products dropped to 12.3 % due to a reclassification of dental care products. Interestingly, despite this reclassification, catastrophic dental care spending did not increase markedly. However, the reclassification is evident when looking at OOPE across all households rather than those experiencing CHE. The share of spending on dental care increased from 7.1 % in 2009/10 to 27.6 % in 2014/15, while the share for medical products decreased from 44 % to 21.3 %.

In 2019/20, dental care emerged as the largest category, representing 25.1 % of OOPE, surpassing outpatient care (23.2 %), diagnostic tests (17.6 %), medical products (14.7 %), medicines (11 %) and inpatient care (8.4 %). In terms of change over time, spending on medicines declined, most likely due to the introduction of the spending cap in 2008, while, notably, outpatient care nearly outranks dental care in the latest wave and diagnostic test surged from 2014/15.

Finally, Supplementary Table S6 presents the breakdown of OOPE by type of healthcare across consumption quintiles to support a more detailed understanding of trends over time. In the poorest quintile, there was a notable shift in the composition of OOPE with the share spent on medicines dropping sharply from 46.6 % in 2004/05 to 23.0 % in 2008/09. This share remained relatively stable up to 2019/20. In that year, medical products and dental care also accounted for significant portions of OOPE in the poorest quintile, making up 26.3 % and 26.1 %, respectively.

## 4 Discussion

Over the course of 4 household budget surveys from 2004/05 to 2019/20 the share of Austrian households with catastrophic health spending has increased from 2.1 % to 3.6 %. It is, however, important to note that the figure of the latest iteration of the survey includes only data on the period from June 2019 to March 2020 to avoid a distortion caused by the COVID-19 lockdown. Thus, only 5,319 of a potential 7,139 observations were used.

The regression analysis highlights the influence of demographic and socioeconomic factors on CHE. Age consistently shows a strong association, with households headed by individuals aged 70 and older having higher odds of experiencing CHE compared to those under 60. Sex was also identified as a factor, with females generally showing higher odds of CHE than males. Employment status and educational attainment are also increasingly relevant, as unemployed individuals and those with lower education levels are more likely to encounter CHE in recent survey waves. These results are consistent with studies in other countries [18], [19], [20].

What is not in line with international findings is the upward trend. While in the two earlier periods, Austria’s share of CHE are a bit higher than in Sweden, Spain or Great Britain, but lower than in Germany and France, it shows a considerable upward trend in the two most recent periods, while most of these countries remain stable or improve (see Fig. S7 in the supplementary material). This also holds true for Germany, Spain or France that have an even more unfavorable age-structure and face similar technological and societal trends. So while potential factors contributing to rising CHE include a higher willingness to pay for medical services, demographic factors and new technologies, these do not seem to be a sufficient explanation.

The rise in CHE from 2004/5 to 2019/20 is also a bit surprising given the generous nature of the Austrian health system and reforms aimed at improving breadth and depth of coverage [6], [21]. Moreover, unlike in most European SHI systems, service coverage in Austrian SHI funds is not restricted to a positive list. Instead, insurees are entitled to legally challenge what should be reimbursed in their individual case, regardless of defined benefits baskets (§ 133 (2) General Social Insurance Act *(“ASVG”))*, and with legal costs of litigation borne by SHI funds to avoid disadvantaging individuals with low incomes.

Additionally, specific policy changes have aimed to broaden SHI coverage. For instance, the 2005 Basic Care Act expanded insurance coverage for refugees and asylum seekers, while the ASVG Amendments Acts of 2010 extended coverage to non-standard employment relationships, e.g., part-time workers, quasi-freelancers, newly self-employed and temporary agency workers [22]. These policies contributed to achieving nearly universal population coverage, with only about 0.01 % of the population – primarily unregistered asylum seekers and students too old to be covered as dependents – remaining uninsured [23]. Further measures to reduce financial burdens, such as the prescription fee cap *(“Rezeptgebührenobergrenze”)* introduced in 2008 and the inclusion of dental braces for children and adolescents as benefits since 2015, also aimed to ease costs for individuals [22].

So what, then, may be the reason for this concerning development? Despite Austria’s extensive SHI coverage, certain services remain only partially reimbursed, contributing to OOPE and financial burdens for households, and the system never seemed to have the strength for a fundamental reform.

Dental care represents the largest share of OOPE in households with CHE. While theoretically, all basic curative services are covered, gaps remain. For instance, dental implants and dental prophylaxis are only covered partially, not at all or only for certain population groups (e.g., children and adolescents). While mercury amalgam fillings are covered, higher-quality alternatives are not. With the planned phase-out of mercury amalgam in January 2025 [24], patients may lose (full) coverage for fillings unless an agreement between SHI funds and the Austrian Dental Chamber is reached.

Outpatient services show the sharpest increase of all OOPE components in households with CHE. Longer waiting times and increasingly shorter consultations at contracted physicians’ have led patients to seek care from non-contracted providers. This trend is mirrored in the growing number of non-contracted physicians, which grew from 6,592 in 2004 to 10,620 in 2019, while the number of contracted physicians remained stagnant at approximately 8,100 in the same period [25], [26]. Evidence suggests that patients willing to pay out-of-pocket for non-contracted providers or make informal payments can secure faster appointments, further raising concerns about equity [27]. With non-contracted physicians, SHI reimburses only up to 80 % of their own fees − typically, fees charged by non-contracted physicians are much higher, resulting in de facto user-charges that above all remain unregistered. This same reimbursement system also applies to some other services like psychotherapy, physiotherapy or midwives.

The sharp increase in spending for diagnostic services among households experiencing CHE in 2014/15 is unexpected, as no relevant policies were implemented in this period. Technological advancements may have contributed to this trend but there is also evidence of long waiting times for SHI-covered MRI scans with shorter waiting times offered to patients willing to pay out-of-pocket [28], [29]. According to a recent survey, 70 % of respondents opted for this alternative [28]. However, there is also evidence of high regional variation of MRI use, much of which remains unexplained by regional characteristics, suggesting potential misuse [30].

The problem in outpatient care and imaging services is exacerbated by slow reform speed with poor integration of care and predominantly single practices. An initiative to establish multi-specialist practices that could efficiently, for example, deliver one-stop diabetes check-ups was thwarted by the Chamber of Physicians in 2008, rekindled in 2017 and 2024 federal agreements, but is still missing concrete legislation [10]. Such inability to structural reform leads to inefficiencies that tie up resources in the public health system, pushing people to seek care outside, incurring high OOPE.

On the plus side, the prescription fee cap introduced in 2008 at 2 % of an individual’s annual net income, seems to have kept OOPE for medicines in check. The share of OOPE on medicines in households with CHE in the poorest quintile has decreased from 47 % in 2004/05 to 23 % in 2009/10 and remained around that level until 2019/20. While this represents a substantial reduction, a considerable burden remains.

One reason may be that many prescribed medicines cost less than the prescription fee, leading patients to purchase them privately at a lower price, which then does not count toward the annual cap. This loophole in the prescription fee cap mechanism has been closed in 2025 by the new coalition government, which also intends to lower the cap to 1.5 % of net annual income by 2030 [31].

However, while the cap has helped limit OOPE for medicines, it does not apply to other types of healthcare such as medical products. This is a significant gap, as medical products accounted for the largest share of OOPE among the poorest quintile in 2019/20.

Some individuals are protected through a different mechanism, which automatically exempts them from paying the prescription fee. This exemption applies to certain vulnerable groups, such as pensioners with low pensions and recipients of social security. Importantly, it also comes with additional benefits, including the removal of co-payments for medical products and exemption from the fixed daily fee for inpatient hospital stays.

Extending similar benefits to those who reach the annual prescription fee cap could provide more comprehensive financial protection. In particular, by covering medical products, this approach could help reduce the overall burden of OOPE among poorer households and consequently reduce CHE.

### 4.1 Strengths and limitations

A major strength of this study is the use of nationally representative household budget survey data across four survey waves from 2004/05 to 2019/20. This enables the analysis of trends in CHE over time and the identification of key socioeconomic factors associated with it. Additionally, the comprehensive nature of the household budget survey data allows for detailed breakdowns of (out-of-pocket) spending categories, offering insights into the drivers of financial hardship in Austrian households.

However, several limitations should be acknowledged. In the 2019/20 household budget survey there is an overlap with the onset of the COVID-19 pandemic. Using the complete dataset, which includes the COVID-19 period, theoretically introduces an upward bias in the CHE estimates and this was also confirmed empirically. To address this, we focused on observations conducted before the pandemic period to mitigate such influences. However, this decision introduces several other limitations. First, household budget surveys are conducted year-round to account for seasonal variations, and by restricting the dataset to June through March, we may have excluded effects specific to the March–June period, potentially biasing the results. Second, the pre-COVID-19 sample may have been constructed in a way that over- or underrepresented specific socioeconomic groups, introducing a potential source of bias. Third, some households may have anticipated COVID-19-related disruptions following early reports of the virus in China and adjusted their purchasing behavior accordingly. While using only 2019 data could have mitigated this issue, we refrained from doing so to avoid further reducing the dataset's size.

Another limitation pertains to inconsistencies in the categorization of household expenses between survey rounds. In 2014/15, dental products were reclassified from “medical products” to “dental care.” While this does not affect the overall CHE estimates, since these expenses remain classified as OOPE, it complicates the comparability of the OOPE composition across waves.

## 5 Conclusion

Despite Austria’s comprehensive SHI system and reforms extending breadth and depth of coverage, the share of households experiencing CHE has increased from 2.1 % to 3.6 % between 2004/05 and 2019/20, and the increase seems to be owed largely to capacity shortages and inefficiencies in publicly funded outpatient care and diagnostics. This begs the question why these gaps remain despite political declarations to the contrary?

One reason seems to be that policies implemented still leave gaps that hurt the increasing number of older and disadvantaged people. An example is the prescription fee cap introduced in 2008 that should probably have been extended to comprise all user charges when the effectiveness of this instrument became clear. Another reason is, and this is also for others to learn from, that Austria is very slow in reforming its healthcare system. While Austrian health policy has good intentions with regard to financial protection, intended policies remain abstract, never come to fruition or are watered down in discussions among the numerous veto players. The vain attempts of SHI’s umbrella organization to harmonize cost sharing, the mantra of strengthening in-kind benefits – repeated since 2017 but without tangible results – together with untackled structural problems that weaken the public system lead to a growing number of services performed in the private sector, leaving people with considerable de facto user charges not even registered by SHI.

The following conclusions can be drawn from this case study. First, financial protection policies must encompass all sources of OOPE, even those occurring outside the public system. As these are frequently unregistered, studies such as this one are valuable sources of information, and the success of policies in one area can inform their expansion to other areas. Second, the continuous adaptation of the system in general improves financial protection, while failure to do so worsens it. Third, good intentions are not enough. Health policy makers need to be tenacious over the whole sequence from analysis and policy design over legislation and implementation, lest the most vulnerable pay the price of inaction.

## 6 Data availability

The datasets used during the current study are available from Statistics Austria upon request. The STATA code can be provided by the corresponding author on reasonable request.

## CRediT authorship contribution statement

**Christoph Stegner:** Writing – review & editing, Writing – original draft, Visualization, Validation, Software, Methodology, Investigation, Formal analysis, Data curation. **Thomas Czypionka:** Writing – review & editing, Writing – original draft, Validation, Supervision, Project administration, Methodology, Investigation, Funding acquisition, Conceptualization.

## Funding

Funding for this study was provided by the WHO Barcelona Office for Health Systems Financing.

## Declaration of competing interest

The authors declare the following financial interests/personal relationships which may be considered as potential competing interests: Thomas Czypionka reports financial support was provided by WHO Regional Office for Europe. Christoph Stegner reports financial support was provided by WHO Regional Office for Europe. If there are other authors, they declare that they have no known competing financial interests or personal relationships that could have appeared to influence the work reported in this paper.
