# Supplementary material for: Good intentions and the costs of inaction: Financial protection in Austria
Source: Health Policy Open. 2025 Dec 16;10:100159. doi: 10.1016/j.hpopen.2025.100159 (PMC12775998; doi:10.1016/j.hpopen.2025.100159)
Supplement: Supplementary Data 1 [file mmc1.docx]

**Supplementary material**

1. Table S1: Sociodemographic characteristics of all households and those exceeding the 40% and 20% threshold of CHE
2. Table S2: Share of households with CHE at the 20% threshold
3. Table S3: Share of households with CHE at the 20%, 25%, 30% and 40% thresholds
4. Table S4: CHE incidence and distribution among consumption quintiles
5. Table S5: Adjusted Odd Ratios of factors associated with CHE from logistic regression analysis
6. Table S6: Breakdown of OOPE by type of health care among households affected by CHE across consumption quintiles (40% threshold)
7. Figure S7: International comparison of the share of households experiencing CHE in selected European countries

Table S1: Sociodemographic characteristics of all households and those exceeding the 40% and 20% threshold of CHE

|  | **2004/05** | | | | **2009/10** | | | | **2014/15** | | | | **2019/20** | | | |
| --- | --- | --- | --- | --- | --- | --- | --- | --- | --- | --- | --- | --- | --- | --- | --- | --- |
|  | **Total (n)** | **Total (%)** | **40% threshold (%)** | **20% threshold (%)** | **Total (n)** | **Total (%)** | **40% threshold (%)** | **20% threshold (%)** | **Total (n)** | **Total (%)** | **40% threshold (%)** | **20% threshold (%)** | **Total (n)** | **Total (%)** | **40% threshold (%)** | **20% threshold (%)** |
| Male | 5406 | 64 | 47 | 53 | 4267 | 65 | 57 | 57 | 4642 | 65 | 53 | 56 | 3102 | 58 | 58 | 52 |
| Female | 2988 | 36 | 53 | 47 | 2264 | 35 | 43 | 43 | 2516 | 35 | 47 | 44 | 2217 | 42 | 42 | 48 |
| 0-59 years | 5777 | 69 | 36 | 40 | 4543 | 70 | 44 | 50 | 4723 | 66 | 42 | 46 | 3459 | 65 | 51 | 47 |
| 60-69 years | 1210 | 14 | 10 | 20 | 973 | 15 | 16 | 18 | 1103 | 15 | 17 | 17 | 848 | 16 | 11 | 13 |
| 70+ years | 1408 | 17 | 54 | 40 | 1015 | 16 | 40 | 33 | 1332 | 19 | 41 | 37 | 1012 | 19 | 38 | 40 |
| Single |  |  |  |  | 1704 | 26 | 21 | 22 | 1988 | 28 | 16 | 19 | 1441 | 27 | 16 | 21 |
| Married |  |  |  |  | 3016 | 46 | 40 | 46 | 3220 | 45 | 46 | 47 | 2575 | 48 | 55 | 49 |
| Other |  |  |  |  | 1811 | 28 | 39 | 33 | 1950 | 27 | 38 | 34 | 1302 | 24 | 29 | 30 |
| Employed | 5027 | 60 | 29 | 36 | 4022 | 62 | 39 | 42 | 3967 | 55 | 27 | 36 | 3157 | 59 | 40 | 39 |
| Unemployed | 236 | 3 | 1 | 1 | 237 | 4 | 3 | 3 | 377 | 5 | 7 | 4 | 238 | 4 | 7 | 5 |
| Retired | 2799 | 33 | 64 | 59 | 2114 | 32 | 56 | 52 | 2397 | 33 | 57 | 52 | 1716 | 32 | 48 | 51 |
| Other | 332 | 4 | 5 | 4 | 158 | 2 | 2 | 3 | 418 | 6 | 9 | 7 | 208 | 4 | 5 | 5 |
| Lower education | 1802 | 21 | 36 | 33 | 1112 | 17 | 35 | 25 | 1153 | 16 | 33 | 25 | 745 | 14 | 32 | 23 |
| Secondary education | 4946 | 59 | 50 | 52 | 4166 | 64 | 58 | 61 | 4819 | 67 | 58 | 63 | 3518 | 66 | 60 | 65 |
| Higher education | 1010 | 12 | 6 | 8 | 1253 | 19 | 7 | 15 | 1185 | 17 | 8 | 12 | 1056 | 20 | 8 | 12 |
| Prefer not to say | 636 | 8 | 8 | 7 |  |  |  |  |  |  |  |  |  |  |  |  |
| High population density | 3355 | 40 | 38 | 42 | 2547 | 39 | 36 | 38 | 2332 | 33 | 31 | 31 | 1820 | 34 | 42 | 35 |
| Intermediate population density | 1984 | 24 | 19 | 20 | 1638 | 25 | 22 | 24 | 1953 | 27 | 21 | 25 | 1635 | 31 | 27 | 30 |
| Low population density | 3055 | 36 | 43 | 37 | 2346 | 36 | 43 | 37 | 2873 | 40 | 48 | 44 | 1865 | 35 | 31 | 35 |
| Single-person household | 2895 | 34 | 47 | 44 | 2332 | 36 | 43 | 40 | 2661 | 37 | 39 | 40 | 1999 | 38 | 31 | 37 |
| 2-person household | 2419 | 29 | 29 | 32 | 1868 | 29 | 23 | 33 | 2137 | 30 | 26 | 30 | 1636 | 31 | 29 | 34 |
| 3/4-person household | 2552 | 30 | 21 | 18 | 1890 | 29 | 20 | 19 | 1911 | 27 | 23 | 22 | 1358 | 26 | 27 | 23 |
| 5+ person household | 528 | 6 | 4 | 5 | 441 | 7 | 14 | 8 | 450 | 6 | 11 | 8 | 326 | 6 | 13 | 7 |
| 0 children up to 13 years | 6625 | 79 | 89 | 87 | 5290 | 81 | 83 | 86 | 5879 | 82 | 79 | 85 | 4351 | 82 | 82 | 87 |
| 1 or 2 children up to 13 years | 1634 | 19 | 10 | 10 | 1138 | 17 | 13 | 11 | 1142 | 16 | 18 | 14 | 852 | 16 | 13 | 11 |
| 3 or more children up to 13 years | 134 | 2 | 1 | 2 | 103 | 2 | 4 | 2 | 137 | 2 | 4 | 2 | 116 | 2 | 6 | 2 |

Figure S2: Share of households with CHE at the 20% threshold


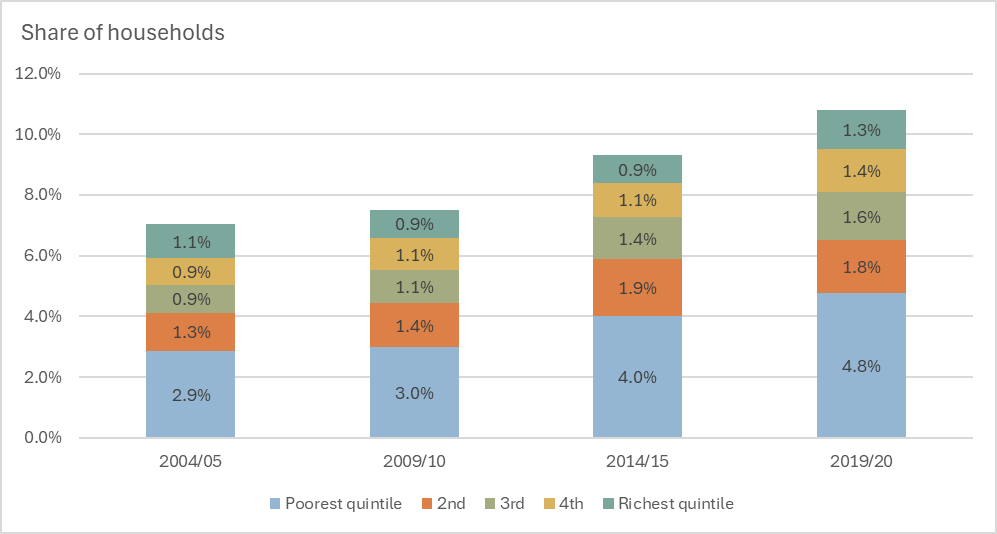


Table S3: Share of households with CHE at the 20%, 25%, 30% and 40% thresholds

|  |  | *Lower CI* | *Upper CI* | **Mean** |
| --- | --- | --- | --- | --- |
| 2004/05 | 20% threshold | *6.3%* | *7.8%* | **7.0%** |
|  | 25% threshold | *4.3%* | *5.4%* | 4.8% |
|  | 30% threshold | *3.0%* | *4.0%* | 3.5% |
|  | 40% threshold | *1.7%* | *2.5%* | **2.1%** |
| 2009/10 | 20% threshold | *6.8%* | *8.3%* | **7.5%** |
|  | 25% threshold | *4.5%* | *5.7%* | 5.1% |
|  | 30% threshold | *3.1%* | *4.1%* | 3.6% |
|  | 40% threshold | *1.7%* | *2.5%* | **2.1%** |
| 2014/15 | 20% threshold | *8.5%* | *10.1%* | **9.3%** |
|  | 25% threshold | *5.8%* | *7.1%* | 6.4% |
|  | 30% threshold | *4.3%* | *5.5%* | 4.9% |
|  | 40% threshold | *2.7%* | *3.7%* | **3.2%** |
| 2019/20* | 20% threshold | *9.8%* | *11.7%* | **10.8%** |
|  | 25% threshold | *6.5%* | *8.2%* | 7.4% |
|  | 30% threshold | *4.9%* | *6.3%* | 5.6% |
|  | 40% threshold | *3.0%* | *4.2%* | **3.6%** |
| 2020** | 20% threshold | *13.7%* | *17.8%* | **15.7%** |
|  | 25% threshold | *10.0%* | *13.6%* | 11.8% |
|  | 30% threshold | *7.6%* | *10.9%* | 9.3% |
|  | 40% threshold | *4.9%* | *7.7%* | **6.3%** |
| 2019/20*** | 20% threshold | *11.1%* | *12.8%* | **12.0%** |
|  | 25% threshold | *7.9%* | *9.5%* | 8.7% |
|  | 30% threshold | *5.8%* | *7.1%* | 6.4% |
|  | 40% threshold | *3.6%* | *4.7%* | **4.2%** |

2019/20* considers only the pre-COVID-19 period, i.e. June 2019 – March 2020
2020** considers only the COVID-19 period, i.e. March 2020 – June 2020
2019/20*** considers the whole period, i.e. June 2019 – June 2020

Table S4: CHE incidence and distribution among consumption quintiles

|  | **Threshold** | **Indicator** | **Poorest** | **2nd** | **3rd** | **4th** | **Richest** | **Total** |
| --- | --- | --- | --- | --- | --- | --- | --- | --- |
| 2004/05 | 20% | Share of all households with CHE | 2.9% | 1.3% | 0.9% | 0.9% | 1.1% | 7.0% |
|  |  | Distribution of CHE households | 40.7% | 17.8% | 13.1% | 12.6% | 15.7% | 100.0% |
|  | 40% | Share of all households with CHE | 1.3% | 0.3% | 0.2% | 0.2% | 0.2% | 2.1% |
|  |  | Distribution of CHE households | 59.5% | 12.6% | 9.2% | 10.3% | 8.3% | 100.0% |
| 2009/10 | 20% | Share of all households with CHE | 3.0% | 1.4% | 1.1% | 1.1% | 0.9% | 7.5% |
|  |  | Distribution of CHE households | 39.8% | 19.1% | 14.7% | 14.0% | 12.4% | 100.0% |
|  | 40% | Share of all households with CHE | 1.2% | 0.3% | 0.3% | 0.1% | 0.2% | 2.1% |
|  |  | Distribution of CHE households | 59.8% | 12.3% | 16.2% | 2.8% | 8.9% | 100.0% |
| 2014/15 | 20% | Share of all households with CHE | 4.0% | 1.9% | 1.4% | 1.1% | 0.9% | 9.3% |
|  |  | Distribution of CHE households | 43.2% | 20.0% | 15.0% | 12.2% | 9.7% | 100.0% |
|  | 40% | Share of all households with CHE | 2.2% | 0.3% | 0.3% | 0.2% | 0.3% | 3.2% |
|  |  | Distribution of CHE households | 69.0% | 9.5% | 8.2% | 5.0% | 8.4% | 100.0% |
| 2019/20* | 20% | Share of all households with CHE | 4.8% | 1.8% | 1.6% | 1.4% | 1.3% | 10.8% |
|  |  | Distribution of CHE households | 44.1% | 16.3% | 14.6% | 13.3% | 11.7% | 100.0% |
|  | 40% | Share of all households with CHE | 2.6% | 0.4% | 0.2% | 0.2% | 0.2% | 3.6% |
|  |  | Distribution of CHE households | 70.8% | 11.7% | 6.3% | 5.3% | 5.8% | 100.0% |
| 2020** | 20% | Share of all households with CHE | 7.7% | 3.0% | 2.3% | 1.5% | 1.1% | 15.7% |
|  |  | Distribution of CHE households | 49.0% | 19.4% | 14.5% | 9.8% | 7.3% | 100.0% |
|  | 40% | Share of all households with CHE | 4.6% | 0.6% | 0.3% | 0.4% | 0.4% | 6.3% |
|  |  | Distribution of CHE households | 73.3% | 9.0% | 5.2% | 6.1% | 6.4% | 100.0% |
| 2019/20*** | 20% | Share of all households with CHE | 5.5% | 2.0% | 1.8% | 1.5% | 1.1% | 12.0% |
|  |  | Distribution of CHE households | 46.1% | 17.1% | 14.7% | 12.6% | 9.6% | 100.0% |
|  | 40% | Share of all households with CHE | 3.0% | 0.4% | 0.3% | 0.3% | 0.2% | 4.2% |
|  |  | Distribution of CHE households | 71.5% | 9.5% | 7.8% | 6.2% | 5.0% | 100.0% |

2019/20* considers only the pre-COVID-19 period, i.e. June 2019 – March 2020
2020** considers only the COVID-19 period, i.e. March 2020 – June 2020
2019/20*** considers the whole period, i.e. June 2019 – June 2020

Table S5: Adjusted Odd Ratios of factors associated with CHE from logistic regression analysis

|  | 2004/05 | | 2009/10 | | 2014/15 | | 2019/20 | |
| --- | --- | --- | --- | --- | --- | --- | --- | --- |
| VARIABLES | 20% threshold | 40% threshold | 20% threshold | 40% threshold | 20% threshold | 40% threshold | 20% threshold | 40% threshold |
| Female (ref=male) | 1.282* | 1.560** | 1.419*** | 1.023 | 1.469*** | 1.526* | 1.324*** | 0.994 |
|  | (0.163) | (0.347) | (0.188) | (0.262) | (0.175) | (0.343) | (0.143) | (0.186) |
| 60-69 years old (ref=0-59 years old) | 2.612** | 0.976 | 1.613 | 2.463 | 1.542** | 1.336 | 1.118 | 0.853 |
|  | (1.194) | (0.386) | (0.474) | (2.103) | (0.323) | (0.525) | (0.292) | (0.419) |
| 70+ years old | 4.610*** | 4.313*** | 3.001*** | 6.085** | 2.900*** | 2.350** | 3.205*** | 2.379* |
|  | (1.823) | (1.638) | (0.881) | (5.074) | (0.624) | (0.825) | (0.898) | (1.126) |
| Unemployed (ref=employed) | 0.459** | 0.672 | 1.300 | 1.084 | 1.265 | 2.854*** | 1.717** | 2.016* |
|  | (0.158) | (0.345) | (0.390) | (0.545) | (0.335) | (1.112) | (0.429) | (0.731) |
| Retired | 0.897 | 1.537 | 1.203 | 1.056 | 1.302 | 2.705*** | 1.436 | 1.696 |
|  | (0.418) | (0.573) | (0.341) | (0.868) | (0.272) | (0.954) | (0.374) | (0.758) |
| Other occupation | 1.124 | 2.029* | 1.615 | 1.260 | 1.728** | 3.197** | 2.206*** | 2.160* |
|  | (0.355) | (0.836) | (0.593) | (0.786) | (0.446) | (1.459) | (0.562) | (0.997) |
| Secondary education (ref=lower education) | 0.874 | 0.970 | 0.919 | 0.690* | 0.790* | 0.639** | 0.734** | 0.467*** |
|  | (0.137) | (0.246) | (0.130) | (0.151) | (0.104) | (0.140) | (0.107) | (0.110) |
| Higher education | 0.712 | 0.738 | 0.732 | 0.270*** | 0.653** | 0.439** | 0.483*** | 0.202*** |
|  | (0.165) | (0.322) | (0.141) | (0.113) | (0.125) | (0.162) | (0.0955) | (0.0749) |
| Prefer not to say | 0.992 | 1.288 |  |  |  |  |  |  |
|  | (0.231) | (0.543) |  |  |  |  |  |  |
| Intermediate pop. density (ref=high pop. density) | 0.767* | 0.808 | 0.926 | 0.827 | 0.872 | 0.710 | 0.812 | 0.571** |
|  | (0.119) | (0.232) | (0.137) | (0.227) | (0.116) | (0.174) | (0.110) | (0.139) |
| Low pop. density | 0.864 | 1.115 | 0.930 | 0.945 | 0.953 | 0.993 | 0.764** | 0.510*** |
|  | (0.129) | (0.244) | (0.127) | (0.224) | (0.123) | (0.223) | (0.100) | (0.114) |
| 2-person household (ref=1-person household) | 1.068 | 1.076 | 1.108 | 0.982 | 0.991 | 1.018 | 1.625*** | 1.662* |
|  | (0.150) | (0.255) | (0.188) | (0.301) | (0.163) | (0.310) | (0.243) | (0.489) |
| 3-4-person household | 0.928 | 1.526 | 1.087 | 1.962* | 1.256 | 1.686 | 2.713*** | 3.917*** |
|  | (0.191) | (0.445) | (0.242) | (0.740) | (0.280) | (0.737) | (0.608) | (1.535) |
| 5-or-more-person household | 1.168 | 1.320 | 2.237** | 7.627*** | 2.141** | 3.223** | 3.932*** | 7.063*** |
|  | (0.317) | (0.553) | (0.732) | (3.760) | (0.671) | (1.798) | (1.387) | (3.703) |
| 1 or 2 children (ref=no children) | 0.950 | 0.773 | 0.805 | 0.800 | 1.042 | 1.549 | 0.568*** | 0.534* |
|  | (0.177) | (0.247) | (0.167) | (0.278) | (0.196) | (0.533) | (0.115) | (0.178) |
| 3 or more children | 2.229* | 0.626 | 1.238 | 1.220 | 0.716 | 1.434 | 0.711 | 1.006 |
|  | (0.992) | (0.561) | (0.508) | (0.655) | (0.304) | (0.912) | (0.344) | (0.617) |
| Married (ref=single) |  |  | 0.984 | 0.528* | 1.297 | 1.320 | 0.792 | 1.052 |
|  |  |  | (0.193) | (0.186) | (0.224) | (0.474) | (0.136) | (0.357) |
| Other marital status |  |  | 0.811 | 0.800 | 0.958 | 1.078 | 0.858 | 1.230 |
|  |  |  | (0.143) | (0.237) | (0.160) | (0.340) | (0.142) | (0.395) |
| Constant | 0.0501*** | 0.00810*** | 0.0531*** | 0.0174*** | 0.0564*** | 0.0114*** | 0.0780*** | 0.0341*** |
|  | (0.0123) | (0.00316) | (0.0123) | (0.00697) | (0.0124) | (0.00469) | (0.0168) | (0.0130) |
| Observations | 8,394 | 8,394 | 6,531 | 6,531 | 7,158 | 7,158 | 5,319 | 5,319 |

std. err. in parentheses

*** p<0.01, ** p<0.05, * p<0.1

Table S6: Breakdown of OOPE by type of health care among households affected by CHE across consumption quintiles (40% threshold)

| **2004/05** | Poorest | 2nd | 3rd | 4th | Richest | Total |
| --- | --- | --- | --- | --- | --- | --- |
| Medicines | 46.6% | 9.4% | 17.1% | 12.6% | 13.5% | 17.0% |
| Medical Products | 31.6% | 56.2% | 22.0% | 21.5% | 15.2% | 23.8% |
| Outpatient Care | 13.7% | 8.9% | 24.6% | 12.4% | 18.2% | 16.1% |
| Dental | 1.7% | 0.0% | 17.6% | 42.1% | 36.9% | 27.7% |
| Diagnostic Tests | 2.4% | 2.2% | 9.7% | 4.5% | 15.6% | 9.7% |
| Inpatient Care | 4.0% | 25.1% | 8.9% | 6.9% | 0.4% | 5.9% |
| **2009/10** | Poorest | 2nd | 3rd | 4th | Richest | Total |
| Medicines | 23.0% | 5.3% | 16.0% | 6.0% | 5.8% | 11.2% |
| Medical Products | 56.4% | 65.9% | 31.0% | 4.3% | 29.5% | 37.2% |
| Outpatient Care | 4.8% | 2.1% | 15.1% | 0.7% | 10.8% | 8.9% |
| Dental | 1.4% | 17.6% | 13.1% | 45.4% | 44.8% | 25.7% |
| Diagnostic Tests | 5.3% | 3.7% | 10.5% | 28.2% | 8.9% | 9.7% |
| Inpatient Care | 9.1% | 5.3% | 14.3% | 15.4% | 0.1% | 7.3% |
| **2014/15** | Poorest | 2nd | 3rd | 4th | Richest | Total |
| Medicines | 24.0% | 17.9% | 12.5% | 12.2% | 7.8% | 13.9% |
| Medical Products | 22.4% | 27.8% | 12.8% | 12.8% | 1.7% | 12.3% |
| Outpatient Care | 6.6% | 9.5% | 22.6% | 40.6% | 11.4% | 15.4% |
| Dental | 35.8% | 14.3% | 37.5% | 13.4% | 25.5% | 26.9% |
| Diagnostic Tests | 1.0% | 8.8% | 11.4% | 15.1% | 29.7% | 16.3% |
| Inpatient Care | 10.3% | 21.7% | 3.3% | 6.0% | 23.8% | 15.1% |
| **2019/20** | Poorest | 2nd | 3rd | 4th | Richest | Total |
| Medicines | 25.4% | 10.9% | 17.1% | 1.4% | 4.5% | 11.0% |
| Medical Products | 26.3% | 24.8% | 15.9% | 8.4% | 4.5% | 14.7% |
| Outpatient Care | 3.8% | 9.6% | 0.0% | 14.0% | 54.8% | 23.2% |
| Dental | 26.1% | 26.2% | 38.7% | 30.0% | 17.0% | 25.1% |
| Diagnostic Tests | 9.9% | 26.5% | 23.8% | 38.2% | 5.6% | 17.6% |
| Inpatient Care | 8.4% | 2.1% | 4.5% | 8.0% | 13.5% | 8.4% |

Figure S7: International comparison of the share of households experiencing CHE in selected European countries

Source: WHO (2024)

**References**

WHO (2024) UHC Watch. WHO Barcelona Office for Health Systems Financing. <https://apps.who.int/dhis2/uhcwatch/#/indicator-explorer>
